# Complete Genome Sequence of Clostridium innocuum Strain ATCC 14501

**DOI:** 10.1128/MRA.00452-20

**Published:** 2020-07-23

**Authors:** K. E. Cherny, E. A. Ozer, T. J. Kochan, L. K. Kociolek

**Affiliations:** a Ann & Robert H. Lurie Children’s Hospital of Chicago, Chicago, Illinois, USA; b Northwestern University Feinberg School of Medicine, Chicago, Illinois, USA; Indiana University, Bloomington

## Abstract

We report the complete genome sequence of Clostridium innocuum ATCC 14501, which was isolated in 1962 from an appendiceal abscess. At that time, the isolated strain was designated C. innocuum, given its suspected lack of virulence, but recent reports suggest that C. innocuum is an emerging pathogen.

## ANNOUNCEMENT

We report the complete genome sequence of Clostridium innocuum strain ATCC 14501, which was identified in 1962 after isolation from an appendiceal abscess (1). It was characterized as a Gram-positive rod with terminal spores. Colonies were 1.5 to 2.5 mm in diameter, glossy, white, raised, and nonhemolytic. Intraperitoneal inoculation of the culture supernatant into mice was nonpathogenic. Since then, C. innocuum has been considered a benign gastrointestinal microorganism (1).

Now, the pathogenic potential of C. innocuum is being reconsidered. Recently, Chia and colleagues reported that C. innocuum is the second most common clostridial species causing extraintestinal infection (2) and is a cause of a Clostridioides difficile-like intestinal infection (3). These isolates were vancomycin resistant, cytotoxic to Vero and HT-29 cells, and enteropathogenic in mice (3). With this reconsideration of C. innocuum pathogenicity, the complete genome sequence of strain ATCC 14501 is needed.

Genomic DNA (gDNA) for both Illumina and Nanopore libraries was extracted using the BiOstic bacteremia DNA isolation kit (Qiagen, Germantown, MD) from an overnight culture grown anaerobically in tryptic soy broth at 37°C. Long-read sequencing libraries were prepared from unsheared gDNA using ligation sequencing kit SQK-LSK109 (Oxford Nanopore, UK) and sequenced on the MinION platform using a FLO-MIN106 flow cell. Default parameters were used for all software unless otherwise specified. Guppy v3.4.5 was used to base call reads with the R9.4.1 high-accuracy model and to perform read quality filtering based on Q scores, demultiplexing, and barcode trimming. Approximate read coverage was 98× from 19,646 reads totaling 460.0 Mbp. The read *N*_50_ value was 22,933 nucleotides, and the *L*_50_ value was 7,784 reads. Nanopore read quality was evaluated using pycoQC v2.5.0.21 (4).

Short-read sequencing libraries were prepared from gDNA using the Nextera XT kit (Illumina, San Diego, CA) and sequenced on the Illumina MiSeq platform using a v3 flow cell to yield 482,327 pairs of 301-bp reads. Sequencing adapters were trimmed from reads on-instrument to generate 196.3 Mbp of sequence, with an approximate genome coverage of 42×. Illumina read quality was evaluated using FastQC v0.11.2 (5). To minimize false-positive variant calls in alignments, no quality trimming of Illumina reads was performed (6).

The genome was assembled with Nanopore reads passing Q score filtering using Flye v2.6 to generate a single 4.30-Mb contig (7). Adapter-trimmed Illumina reads were aligned to the assembly using BWA v0.7.17, and assembly errors were corrected using Pilon v1.23 with a --mindepth setting of 0.1 (8, 9). Serial read alignment and Pilon correction were performed three times until no further assembly corrections were generated. Custom software (Pilon Tools v0.1) (https://github.com/egonozer/pilon_tools) was used to identify, manually assess, and correct any residual homopolymer assembly errors. Circulator v1.5.5 was used to ensure circularization of the chromosome by trimming overlapping ends and rotating to the start of the *dnaA* gene (10). The final assembly is a single chromosomal sequence with a length of 4,718,906 bp and a GC content of 43.6%. Annotation was performed using the NCBI Prokaryotic Genome Annotation Pipeline (PGAP) v4.11 (11, 12).

### Data availability.

This project has been deposited in DDBJ/ENA/GenBank under accession number CP048838. The raw data accession numbers are SRR11552096 and SRR11552095.

## References

[1] Smith LD, King E 1962 *Clostridium innocuum*, sp. n., a spore-forming anaerobe isolated from human infections. J Bacteriol 83:938–939. doi:10.1128/JB.83.4.938-939.1962. 13914326 PMC279386

[2] Chia JH, Feng Y, Su LH, Wu TL, Chen CL, Liang YH, Chiu CH 2017 *Clostridium innocuum* is a significant vancomycin-resistant pathogen for extraintestinal clostridial infection. Clin Microbiol Infect 23:560–566. doi:10.1016/j.cmi.2017.02.025. 28254687

[3] Chia JH, Wu TS, Wu TL, Chen CL, Chuang CH, Su LH, Chang HJ, Lu CC, Kuo AJ, Lai HC, Chiu CH 2018 *Clostridium innocuum* is a vancomycin-resistant pathogen that may cause antibiotic-associated diarrhoea. Clin Microbiol Infect 24:1195–1199. doi:10.1016/j.cmi.2018.02.015. 29458157

[4] Leger A, Leonardi T 2019 pycoQC, interactive quality control for Oxford Nanopore sequencing. Joss 4:1236. doi:10.21105/joss.01236.

[5] Andrews S 2010 FastQC: a quality control tool for high throughput sequence data. http://www.bioinformatics.babraham.ac.uk/projects/fastqc.

[6] Liu Q, Guo Y, Li J, Long J, Zhang B, Shyr Y 2012 Steps to ensure accuracy in genotype and SNP calling from Illumina sequencing data. BMC Genomics 13(Suppl 8):S8. doi:10.1186/1471-2164-13-S8-S8. PMC3535703 23281772

[7] Kolmogorov M, Yuan J, Lin Y, Pevzner PA 2019 Assembly of long, error-prone reads using repeat graphs. Nat Biotechnol 37:540–546. doi:10.1038/s41587-019-0072-8. 30936562

[8] Li H, Durbin R 2009 Fast and accurate short read alignment with Burrows-Wheeler transform. Bioinformatics 25:1754–1760. doi:10.1093/bioinformatics/btp324. 19451168 PMC2705234

[9] Walker BJ, Abeel T, Shea T, Priest M, Abouelliel A, Sakthikumar S, Cuomo CA, Zeng Q, Wortman J, Young SK, Earl AM 2014 Pilon: an integrated tool for comprehensive microbial variant detection and genome assembly improvement. PLoS One 9:e112963. doi:10.1371/journal.pone.0112963. 25409509 PMC4237348

[10] Hunt M, Silva ND, Otto TD, Parkhill J, Keane JA, Harris SR 2015 Circlator: automated circularization of genome assemblies using long sequencing reads. Genome Biol 16:294. doi:10.1186/s13059-015-0849-0. 26714481 PMC4699355

[11] Tatusova T, DiCuccio M, Badretdin A, Chetvernin V, Nawrocki EP, Zaslavsky L, Lomsadze A, Pruitt KD, Borodovsky M, Ostell J 2016 NCBI Prokaryotic Genome Annotation Pipeline. Nucleic Acids Res 44:6614–6624. doi:10.1093/nar/gkw569. 27342282 PMC5001611

[12] Haft DH, DiCuccio M, Badretdin A, Brover V, Chetvernin V, O'Neill K, Li W, Chitsaz F, Derbyshire MK, Gonzales NR, Gwadz M, Lu F, Marchler GH, Song JS, Thanki N, Yamashita RA, Zheng C, Thibaud-Nissen F, Geer LY, Marchler-Bauer A, Pruitt KD 2018 RefSeq: an update on prokaryotic genome annotation and curation. Nucleic Acids Res 46:D851–D860. doi:10.1093/nar/gkx1068. 29112715 PMC5753331

